# OBLIGATIONS IN JAPAN: A THREE-YEAR LONGITUDINAL STUDY OF MID-LIFE ADULTS

**DOI:** 10.1093/geroni/igad104.3501

**Published:** 2023-12-21

**Authors:** Jeewon Oh, Han Na Lee

**Affiliations:** Syracuse University, Syracuse, New York, United States; Loyola University Chicago, Chicago, Illinois, United States

## Abstract

Obligations embody a sense of responsibility, whether they are directed toward close relationships or the broader public community. Middle-aged adults may find themselves in increased caregiving roles, but research examining the role of their obligation on their well-being and relationships is scarce. Across studies, obligation is linked with both positive (e.g., higher well-being and relationship quality) and negative outcomes (e.g., greater burden). A previous study in the U.S. suggested that these mixed findings may be partially because there are various types of obligations that differ on the level of investment. Given the Japanese collectivistic culture that values intricate social connection, their obligation may take on a different form and play a different role. We used two waves of data from the Midlife in Japan Project (2009-2012). Participants were 371 middle-aged adults (M = 55.47, SD = 14.04, 56.02% women, 43.98% men, 71.73% married) living in Japan. We factor-analyzed the structure of obligation and found a 3-factor solution fit best (e.g., RMSEA = .06, SRMR = .01, CFI = 1.00). Obligation could be characterized by holding light and substantive obligations to close others and obligations to the public community. Results suggest differential links between the types of obligation and well-being outcomes. For instance, light obligation predicted less negative affect (β = -.24, p = .035) after three years, but otherwise, other types of obligation did not predict positive or negative affect. Links with relational outcomes (e.g., support/strain) and implications of midlife obligations will be discussed.

